# NiMBaLWear analytics pipeline for wearable sensors: a modular, open-source platform for evaluating multiple domains of health and behaviour

**DOI:** 10.1186/s44247-024-00062-3

**Published:** 2024-02-08

**Authors:** Kit B. Beyer, Kyle S. Weber, Benjamin F. Cornish, Adam Vert, Vanessa Thai, F. Elizabeth Godkin, William E. McIlroy, Karen Van Ooteghem

**Affiliations:** 1 Department of Kinesiology and Health Sciences, Faculty of Health, University of Waterloo, Waterloo, ON, Canada.

**Keywords:** Wearable technology, Remote monitoring, Analytics, Multi-sensor, Open-source, Older adults, Neurodegenerative disease, Clinical application, Health-related behaviors, Digital health

## Abstract

**Background:**

Recent technological advances have led to a surge in the use of wearable devices for personal health and fitness monitoring; however, clinical uptake of wearable devices for remote or ‘free-living’ measurement of daily health-related behavior has lagged. To advance the field, there is need for valid and reliable outcomes across multiple health domains specific to the cohorts or patients of interest and centralized tools to build capacity for use of these data. The NiMBaLWear pipeline provides a flexible and integrated approach to wearables analytics applied to raw sensor data that considers multiple, inter-related physiological and behavioral signals to provide a holistic view of health status.

**Results & discussion:**

NiMBaLWear is a modular, open-source, wearable sensor analytic pipeline that quantifies physical activity, mobility, and sleep from raw single- or multi-sensor free-living data collected over multiple days. Data captured from any device, in different possible formats, are standardized prior to processing. Data preparation includes accelerometer autocalibration, cross-device synchronization, and non-wear detection. Validated, domain-specific algorithms detect events, generate outcome measures, and output standardized tabular data and user-friendly summary collection reports. NiMBaLWear was developed in Python using an iterative and incremental software development process, which included a combination of semi-automated inspection and expert review of data collected from 286 participants across two remote-measurement studies. A comparative analysis revealed a paucity of open-source packages capable of deriving and sharing health-related behavioral outcomes across multiple domains from multi-sensor wearables data. Forthcoming improvements to the pipeline will leverage sensor fusion techniques to add new, and refine existing, domain- and disease-specific analytics, and optimize pipeline accessibility and reporting.

**Conclusion:**

The NiMBaLWear pipeline transforms raw multi-sensor wearables data into accurate and relevant outcomes across multiple health domains to objectively characterize and measure an individual’s daily health-related behavior. NiMBaLWear’s focus on high-quality, clinically relevant outcomes, as well as end-user optimization, provides a foundation for innovation to improve the utility of wearables for clinical care and self-management of health.

## Background

Advances in non-invasive wearable and ambient sensor technologies, including developments in sensor networks, communication protocols, and feature classification, have created a landscape of opportunity for the clinical use of health-related information captured in daily life [1–3]. A shift toward remote or ‘free-living’ measurement outside of a clinic or laboratory with digital health technologies, enables monitoring of behaviors and symptoms as they occur throughout the day, reducing reliance on patient self-report [3–5] and minimizing the opportunity for observer effects [6, 7]. Continuous remote measurement can also capture infrequent events that are unlikely to occur under the observation of a healthcare provider [4, 8–10]. With continued advances, clinically relevant information derived from remotely acquired data could positively impact health outcomes and decrease healthcare costs by identifying declines in health that can inform early diagnosis and timeliness of care [11–13]. Unfortunately, while consumer use of wearable technologies (‘wearables’) for health monitoring has grown tremendously – largely driven by the adoption of smartwatches for general health and fitness tracking – clinical uptake has lagged. Integration of wearables into the healthcare system has been limited, in part, by the need for analytical and clinical validation of digital end-points [14, 15] as well as the relative absence of technical, training, and change management processes to support this transition [16, 17]. The current work describes an open-access, easy-to-use analytics pipeline (NiMBaL-Wear) designed to minimize these barriers and maximize the utility of wearables for clinical application.

NiMBaLWear was designed and developed according to four criteria deemed essential for clinically meaningful remote data capture and analysis within our populations of interest, which primarily include older adults and persons living with complex health conditions: 1) multi-domain measurement, 2) device independence, 3) pipeline modularity and extensibility, and 4) pipeline and data usability. NiMBaLWear adopts a flexible and integrated approach to health monitoring that considers multiple, inter-related physiological and behavioral domains (e.g., mobility, sleep, activity, cardiovascular function) to provide a more holistic view of health status.

This multi-domain approach necessitates a multi-sensor model that accommodates a variety of sensor types (‘modes’) worn at appropriate body locations (‘nodes’) to optimize the type and quality of raw data that is used to construct the valid metrics of health required for clinical purposes [4, 10, 18–21]. NiMBaLWear development has prioritized a ‘low-burden’ multi-modal and multi-nodal model not afforded by currently available software that is either limited to processing data from a particular device manufacturer or uses a single-sensor approach. Currently, NiMBaLWear utilizes accelerometer, gyroscope, and temperature sensor data from wrist- and ankle-worn devices across its data preparation and analytics algorithms. However, to accommodate the future integration of additional sensor modes (e.g., electrocardiography (ECG), Global Positioning System (GPS)) and nodes (e.g., thigh, chest), the NiMBaLWear pipeline maintains device independence by supporting data ingestion from various devices and including pre-processing modules to temporally synchronize incoming data. This model makes it possible to unify the measurement and analysis of multiple health domains by optimizing the information captured from individual devices and, when appropriate, fusing data from multiple inputs.

To support a wide range of current and future clinical applications, NiMBaLWear’s modular design allows users to select domains and outcomes based on clinical need and sensor availability. This modularity begets an extensibility that also allows refinement of outcomes in existing domains or the addition of new health domains. Current NiMBaLWear outcomes focus on mobility (e.g., gait), sleep, and activity/sedentary behaviour, which were identified as priorities given their relationship to chronic disease [22–25], their importance for self-management of health [26, 27], and their potential to support early detection of disease [22, 26, 28–31]. Imminent expansions include the addition of full-body posture and cardiac outcomes, disease-specific outcomes, and refinement of existing sleep, mobility, and activity outcomes based on additional sensor inputs.

Importantly, consumer technology typically includes proprietary software, which outputs highly processed data and creates barriers for clinical application due to challenges in understanding, reconstructing, and evaluating the analytics, as needed, for clinical validation [14, 32]. Therefore, NiMBaLWear was designed with a focus on transparency and accessibility, allowing researchers and clinicians with limited programming expertise to process and access data. Pipeline-generated outputs ensure data has utility for foundational work in research and for patient and clinician reporting. The current version of NiMBaLWear is being used in several data collection initiatives acquiring continuous wearable sensor data from over 400 participants to date, most of whom are older adults and/or persons living with neurodegenerative disease.

An essential motivation for the development of the pipeline was to create a platform on which to consolidate best practices and innovation in data analytics for wearable sensors across a range of health domains. This serves, in our view, as a critical phase of development to enable clinical research and inform/evaluate commercial applications that are intended for clinical applications. This paper provides an overview of NiMBaLWear, with an eye toward continued advancement through the collective effort of a community of experts with a shared vision.

## Implementation

### NiMBaLWear overview

NiMBaLWear is an open-source, Python-based [33] data processing pipeline that currently detects and quantifies gait, sleep, and activity from multi-day, multi-device, free-living wearable device data. To accommodate multi-device collection and analysis, NiMBaLWear organizes incoming wearable data collections, devices, and sensors. A data collection is a group of related devices collected simultaneously and attached to the same person. A device is a single physical device that may contain multiple sensors (e.g., accelerometers, gyroscopes, ECG, temperature) but usually outputs data to a single file. The Collection and Device objects are used to store data in memory during processing. For longer term storage, all data, including raw wearable device data, metadata, and output data, are organized within a single study folder in a defined structure.

### Pipeline features

**Fig. 1:**
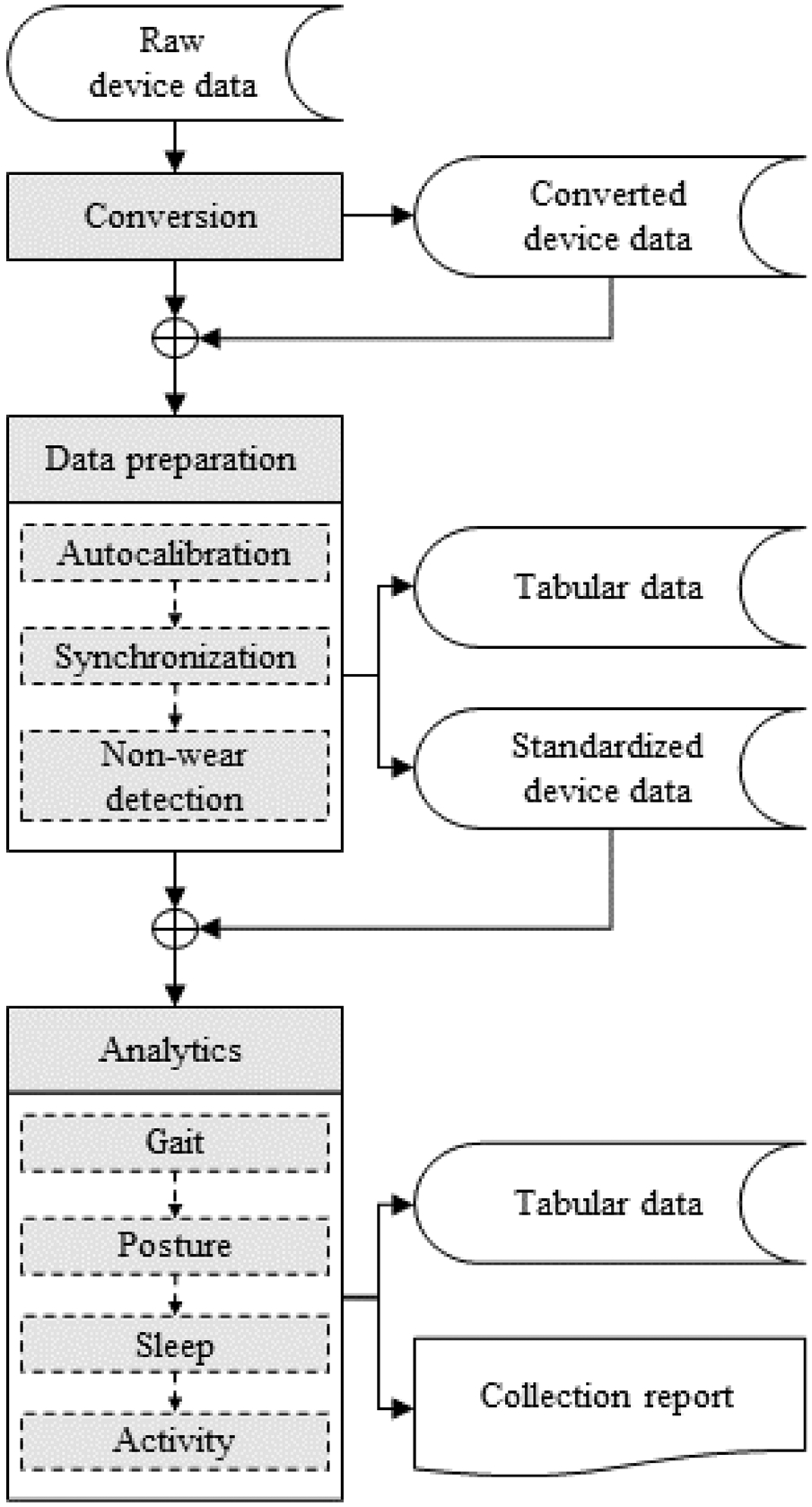
Simplified flow of data through the NiMBaLWear pipeline

NiMBaLWear data processing occurs in three separate stages: 1) conversion, 2) data preparation, and 3) analysis. When run independently, the outputs of each stage are saved to file and can be used in subsequent processes. When run sequentially, the outputs of each stage are saved to file but also held in memory to be used in the subsequent process, so that they do not need to be re-read. For each stage, some default parameters are described below, but most pipeline parameters may be customized via a settings.toml file. An overview of the logic model is presented in Fig. 1.

#### Conversion

The conversion stage imports raw wearable device data and exports to standard European Data Format (EDF) files [34, 35]. NiMBaLWear can convert data from several devices including Axivity (Axivity Ltd., Newcastle Upon Tyne, UK), GENEActiv (Activinsights Ltd., Kimbolton, UK), Bittium (Bittium USA, Bothell, WA), and ActiGraph (ActiGraph, Pensacola, FL). Inclusion of any other device is possible with access to the raw data (e.g., conversion from any proprietary data format structures) and will either require external conversion to an appropriately formatted EDF file or the addition of a module for device-specific conversion. Within NiMBaLWear, EDF file operations are supported by the pyEDFlib package [36].

#### Data preparation

The data preparation stage, including accelerometer autocalibration, device synchronization, and non-wear detection as described below, ensures consistent, high-quality data are prepared for further analysis regardless of the device from which they are collected.

##### Accelerometer autocalibration

The relationship between voltage and acceleration is calibrated using an iterative closest-point fitting process during periods when the accelerometer is not moving so that gravity, a known constant, is the only acceleration being detected [37]. Calibration outputs, such as the amount of calibration error in each axis before and after calibration and the number of iterations performed, are stored to file.

##### Synchronization

Every wearable device experiences unique internal clock drift causing slightly different sampling rates across devices. For multi-day collections, this drift can cause significant temporal misalignment of data from different devices. A paucity of hardware-synchronized devices that permit collection from multiple body segments with access to raw data necessitated an approach to synchronize different devices that takes advantage of the accelerometer sensor included in most products. The current method requires a series of manual synchronization events to be performed with all devices in unison at various timepoints throughout the collection. Each synchronization event consists of 5 to 10 device rotations with 5 to 10 seconds of rest between creating an easily detectable repeating square waveform in the accelerometer. The detected synchronization events are used to resample the data into alignment. Synchronization outputs, including the timing of each sync event in all devices, the calculated clock drift, and subsequent correction, are stored to file.

##### Non-wear detection

Periods of device non-wear are detected using our DETACH algorithm that combines characteristic changes in acceleration and near-body temperature (when available) to detect when a device has been removed or reattached [38]. Specifically, standard deviation of acceleration, rate of temperature change, and absolute temperature are considered over 5-minute rolling windows to determine the start and end of non-wear periods. A classification and regression tree was used to determine the optimal values for these parameters depending on the wear location of the device (e.g., chest, wrist, ankle). Optionally, long periods of non-wear at the beginning and end of collection can be cropped from the device data to remove data collected outside of the intended period, such as during shipment of devices to and from participants. A cropped version of the standardized EDF file is stored if this option is selected. Non-wear outputs, including the timing of each period of wear and non-wear, are stored to file.

#### Domain-specific analytics

The current pipeline modules, described below, focus on inertial measurement unit (IMU) data from wrist- and ankle-worn devices. Currently, in each domain, raw data from a single, most appropriate node is used to detect events and generate outcomes. Importantly, our multi-modal, multi-nodal approach, including cross-device temporal synchronization of raw data, allows cross-domain comparison of events and outcomes derived from different underlying nodes. Continued analytics development (see Planned Future Developments and Intended Use) is further supported by this model by permitting the flexibility to calculate outputs for a specific domain from different sources, depending on the availability of devices and sensors, and allowing fusion of data from multiple sensors. NiMBaLWear’s modular design also supports these analytic advances by permitting simple integration of additional modules.

##### Gait

Gait – including walking, jogging, and running – is detected using gyroscope or accelerometer data from an ankle-worn device. Candidate steps can be identified using either a gyroscope- or accelerometer-based algorithm before combining temporally proximal steps into gait bouts. The gyroscope-based step detection algorithm detects positive angular velocity peaks that occur above an adaptive threshold – these peaks correspond with the mid-swing point of each candidate step [39]. This algorithm also detects other rhythmic lower limb movements, such as cycling, that can be manually removed to improve step identification [40]. However, by default, these movements are retained so that broad measures of activity (step count and intensity minutes) remain closely aligned. The accelerometer-based step detection algorithm employs a finite state machine algorithm to identify gait phases using the vertical axis acceleration – the local negative peak of each swing phase corresponds with the mid-swing point of each candidate step [41]. Groups of at least two candidate steps occurring within two seconds of the previous step are combined into gait bouts [42–44]. Candidate steps that do not occur within a defined gait bout are not included for further analysis. Gait outputs, including the timing of detected steps and gait bouts, as well as the number of steps within each gait bout, are saved to file.

##### Sleep

Sleep is detected using wrist accelerometer data by first identifying sleep period time windows (SPTWs) during which sleep is likely to occur and then searching within these windows for sleep bouts. SPTWs are detected by assessing z-axis angle variance, where the z-axis is positioned perpendicular to the skin surface, as previously described [45]. All SPTWs greater than 30 minutes are retained and those occurring within 60 minutes of each other are combined. Within each SPTW, sleep is detected when there is no change in z-axis angle greater than 5 degrees for at least 5 minutes [46]. SPTWs that do not contain sleep bouts are removed from further analysis. Each SPTW and sleep bout is defined as overnight sleep if it overlaps the customizable overnight period (10 pm to 8 am, by default). All other SPTWs and sleep bouts are defined as daytime sleep. Sleep outputs, including the timing of each SPTW and sleep bout, and measures of sleep duration, sleep-to-wake duration, sleep efficiency, and wake after sleep onset, are saved to file.

##### Activity and sedentary behaviour

Activity intensity classification is performed using wrist accelerometer data. Accelerometer data is filtered using a 20 Hz low-pass filter [47] and the average vector magnitude (AVM) of acceleration is then calculated in 15-second epochs. Epochs that do not overlap with detected non-wear or sleep are classified as sedentary, light, moderate, or vigorous intensity by comparing the AVM to epoch length-independent activity intensity cut-points, which may be derived from epoch length-dependent cut-points, if necessary [47]. NiMBaLWear allows cut-points to be customized based on participant age and, by default, uses separate published cut-points for participants under 60 years old [48] and for those 60 years and older [47]. Activity bouts are then constructed by combining consecutive epochs of the same intensity. Activity outputs, including timing and AVM of each epoch, and timing and intensity of activity bouts, are saved to file.

### Pipeline outputs

**Fig. 2:**
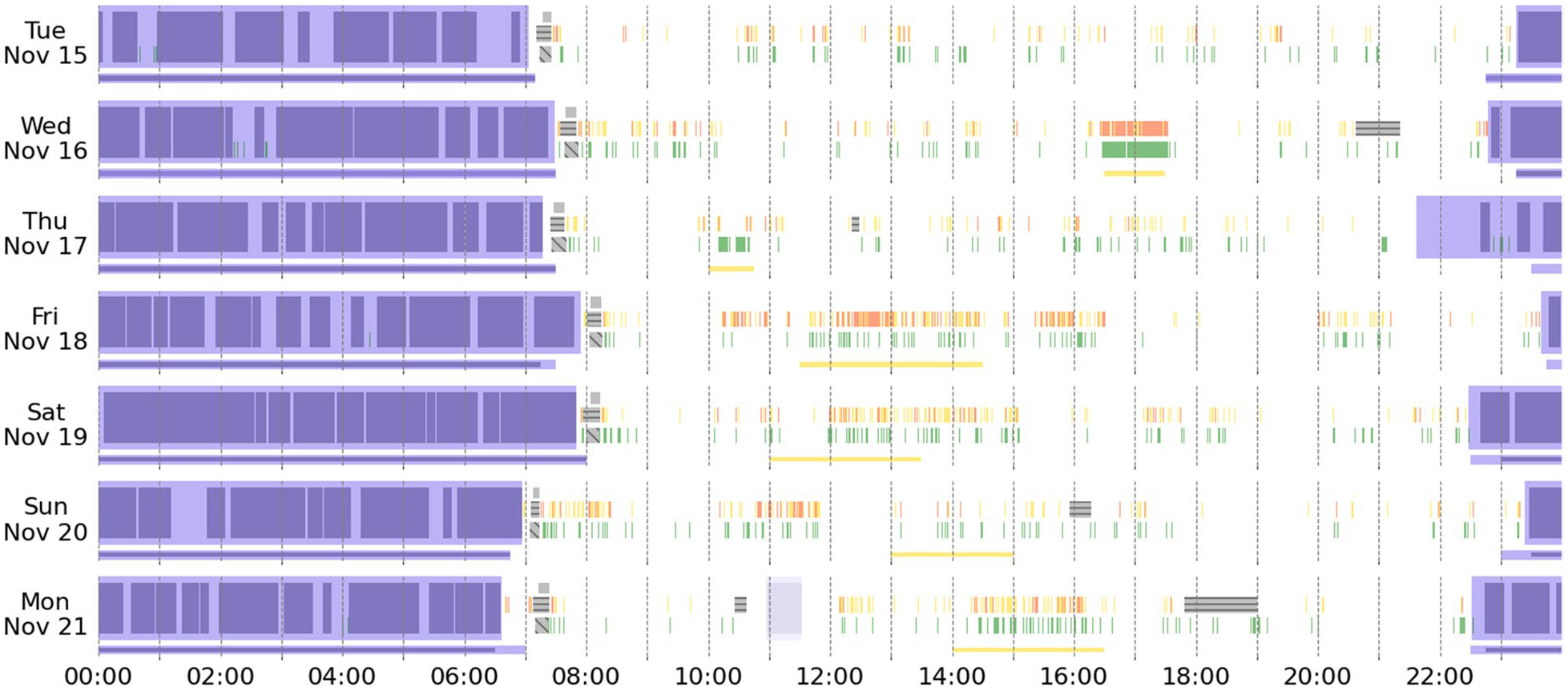
Sample collection report. Data is included from multiple sensors to integrate information about sleep (light purple = SPTW; deep purple = sleep bouts), gait (green), activity intensity (yellow = light; orange = moderate-to-vigorous), and non-wear (grey, with hatching indicating specific device), within and across days, for each participant. Each row represents a 24-h period (days 1–7 stacked), from midnight to midnight. Duration of the specific behavior is represented by the width of the block. For each day, participant self-reported sleep and activity are displayed as thin bars below the sensor-derived, pipeline-generated data

The pipeline automatically generates several categories of outputs including: 1) standardized raw data that is synchronized in time against all other devices in the data collection, 2) tabular data for outcome measures from the respective domain-specific analytics, and 3) the summary collection report for visualizing core analytic outputs presented in time across all days (Fig. 2). These standardized outputs facilitate the development of supplemental, user-generated reports that can serve a variety of purposes. For example, recent work by our group utilized NiMBaLWear outputs to generate a feedback report designed to provide study participants with information to guide self-management of daily health-related behaviors [49].

#### Standardized device data

For each device that is processed using the pipeline, a raw device EDF is output after the conversion stage and a *standardized* device EDF is output after the auto-calibration and synchronization processes are complete. If cropping is selected, a cropped device EDF is output following non-wear detection. By default, the pipeline saves and uses a single EDF for each device, but there is an option to create a separate EDF file for each sensor type within a device (e.g., accelerometer, gyroscope, temperature).

#### Tabular data

Tabular data, including the timing of detected events, device- and event-specific analytics, and summary outcomes, are output in standard format, comma-separated values (CSV) files. All outputs contain common fields identifying the study, participant, and collection to allow outputs from multiple collections, participants, and studies to be combined for further analysis. Device-specific outputs (e.g., non-wear, calibration, synchronization) contain fields identifying the device type and location of wear. Event-specific outputs contain an event label and identification number as well as start and end timestamps, if applicable. Event timestamps directly correspond with timestamps of the synchronized device data. Specific outputs of each pipeline stage have been noted above (see Domain-specific analytics) and are summarized in Table 1.

#### Summary collection report

The collection report is an automatic output from the pipeline used to visually inspect synchronization, domain-specific analytics, and detected non-wear periods for all devices (Fig. 2). The integrated presentation of data within the collection report is particularly useful for identifying potential data irregularities. It is used to visualize the data immediately after processing, providing an overview of the various outcome measures in time across days. The collection report also includes a summary of digitally stored, participant-reported information generated in an annotated text file which, depending on the study, can also include timestamps of logged activities or events (e.g., sleep, activity, medication timing).

#### Pipeline logs

Every time the pipeline is used to process a data collection, a log file is generated. The log contains a header that includes the study, participant, and collection identifiers, the pipeline stages included, the version of NiMBaLWear used, and all customizable parameters used for each stage. This information is followed by a series of messages generated as the data moves through the pipeline. Each message is labelled with the date and time, and its level of importance: INFO, WARNING, or ERROR. INFO messages relay information about the processes performed during each stage and substage of the pipeline. WARNING messages indicate that something unexpected occurred, but the pipeline was able to complete its operation. WARNING messages may indicate issues with the data inputs or outputs and should be investigated further. ERROR messages indicate that an exception occurred that prevented the pipeline from completing the operation. When an ERROR occurs, the exception traceback information is exported to the log and processing of that data collection is aborted.

### Pipeline usage

**Fig. 3:**
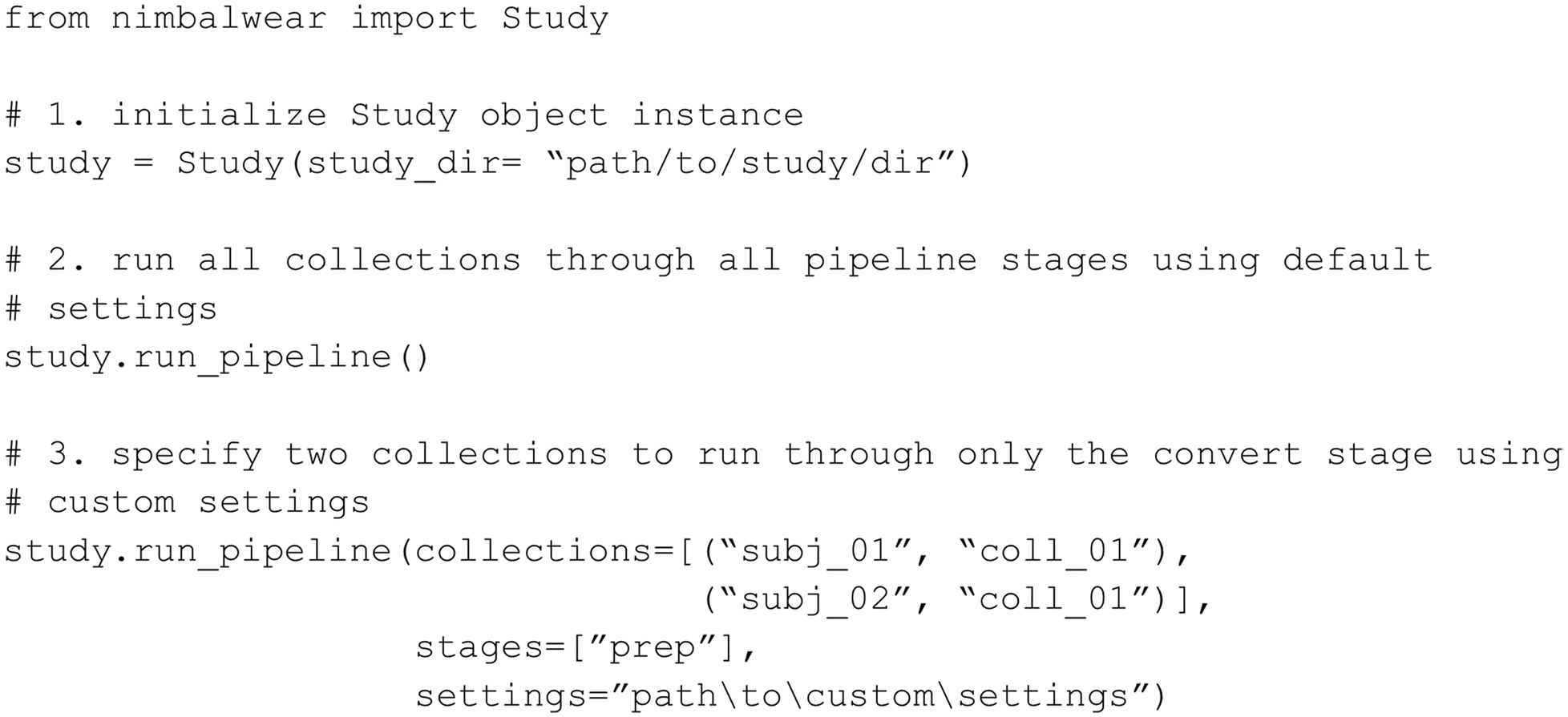
Sample usage script. In this script, (1) initializes a Study object, (2) runs all available data collections through all stages of the pipeline using the default parameters, and (3) runs two specific data collections through only the data preparation stage of the pipeline using custom parameters

Figure 3 demonstrates a simple usage example for processing data with NiMBaLWear. Prior to executing the pipeline, required study data needs to be organized in a study directory. The required data include all raw device data files and two CSV files containing information about the data collections and devices included in the study. Detailed instructions describing how to organize data and specify custom parameters before executing the pipeline can be found in the documentation included with the software. Following data preparation, processing can proceed by initializing a *nimbalwear.Study* object with the path to the study folder and executing the *Study. run_pipeline* method with the option to specify which collections to include, which stages and to perform, and any custom settings that should be used.

### Development and evaluation

NiMBaLWear was developed in Python using an iterative and incremental software development process and data from two remote measurement studies (Remote Monitoring in Neurodegenerative Disease (ReMiNDD) and Health in Aging, Neurodegenerative Diseases and Dementias in Ontario (HANDDS-ONT)) deployed within the Ontario Brain Institute (OBI) Ontario Neurodegenerative Disease Research Initiative (ONDRI) Integrated Discovery Program (IDP). In ReMiNDD, 39 community-dwelling adults living with cerebrovascular or neurodegenerative disease wore devices on both ankles, both wrists, and the chest for one week [50]. Each wrist and ankle device (GENEActiv Original) collected accelerometer and temperature sensor data. The chest device (Bittium Faros 180) collected ECG and accelerometer sensor data. In HANDDS-ONT, 247 community-dwelling adults, with or without neurodegenerative disease (approximately 50% per cohort), wore devices on one wrist, one ankle, and the chest for 10 days. Each wrist and ankle device (Axivity AX6) collected accelerometer, gyroscope, and temperature sensor data. The chest device (Bittium Faros 360) collected ECG, accelerometer, and temperature sensor data. The human research ethics committee of Sunnybrook Research Institute, Toronto, ON, Canada, approved both the ReMiNDD (REB approval: 007–2019) and HANDDS-ONT (REB approval: 2021–1517) studies, and all participants provided written informed consent before participating.

A minimum viable product (MVP) was developed using data from the ReMiNDD study. The MVP included data conversion to standardized EDF for GENEActiv and Bittium files and detected non-wear from accelerometer data only. Domain-specific analytics (gait, sleep, and activity) were performed using a single device for each. The pipeline was then evaluated and improved over a two-year period coinciding with the HANDDS-ONT data collection. This included iterative quality control processes to evaluate data integrity, as well as expert data review of the raw data, tabular data, and collection reports for all study participants. Errors and inefficiencies identified through this process led to several revisions and adaptations as described below. The process of evaluation, improvement, and continued development is ongoing Priority targets for future work are detailed below.

## Results and Discussion

### NiMBaLWear pipeline performance evaluation

Key evaluation outcomes established using data from the HANDDS-ONT study are presented below alongside illustrative data, where appropriate, to demonstrate the effect of algorithm modifications on underlying data quality.

#### Gyroscope-based gait algorithm

The MVP was developed using an accelerometer-based gait algorithm, but inclusion of a gyroscope in HANDDS-ONT permitted evaluation of a gyroscope-based gait detection algorithm. Visual inspection of accelerometer data alongside detected gait bouts and identified step locations during data review of the first 20 participants revealed fewer errors in gait detection for the gyroscope-versus accelerometer-based algorithm, particularly in individuals with slower gait (i.e., lower acceleration and angular velocity magnitudes during stepping). Both algorithms are included as options to account for equipment/sensor availability and different gait features across cohorts.

#### Activity classification improvements

Several improvements to activity classification were implemented after expert data review. Visual inspection of wrist-derived activity classification alongside ankle-derived gait bout data revealed the occurrence of gait bouts that were being classified as sedentary, which we have reported in detail elsewhere [51]. Further inspection of raw wrist accelerometer and ankle gyroscope data revealed two separate causes for this discrepancy. First, in many older adults, the wrist movement associated with arm swing did not reach commonly used cut-points for light intensity activity [48]. Upon further review, it became clear that the single set of cut-points used to classify activity intensity was not appropriate for the wide age range of HANDDS-ONT participants (mean: 65.6 ± 12.3 years, range: 20–92 years). To address this discrepancy, we added the option to define customizable age-based activity intensity cut-points and, by default, included separate cut-points for adults under 60 years old [48] and those 60 years and older [47]. Second, during some gait bouts, no arm swing was detectable from the wrist accelerometer. Participant activity logs revealed instances of daily life activity (e.g., walking a dog, stationary cycling) that could be characterized by identifiable stepping (or cycling) activity without significant arm movement. To mitigate such classification errors, a separate activity classification was created using gait data from the ankle sensor to distinguish epochs classified as sedentary that coincided with gait from those that did not. Across 122 participants for which this distinction was made, 9.6% of total sedentary time occurred during gait. Currently, we manage these instances by excluding them from activity classification. Ongoing work will provide the option to further refine activity classification based on gait status, full-body posture (i.e., lying vs. sitting vs. standing), and heart rate when appropriate devices are available.

#### Cross-device synchronization

While activity and gait outcomes are separately derived from a single device (wrist and ankle, respectively), the cross-domain comparison required to identify periods of sedentary classification that overlapped with gait bouts required close temporal synchronization between devices. In anticipation of the need for cross-device synchronization, HANDDS-ONT included a series of synchronization events performed throughout the data collection, as described above. We first developed an algorithm to detect these events in each device separately. Comparison of the timing of 638 synchronization events across 126 participants revealed a mean (SD) absolute clock drift rate of 2.51 (3.81) seconds per day. To correct for this drift, the algorithm was modified to resample data between detected synchronization events to bring them into alignment.

#### Accelerometer autocalibration

Review of activity classification data for early participants suggested total activity was overestimated in some cases. Specifically, six of the first 55 participants averaged more than 400 min of total activity per day. Visual inspection of wrist accelerometer vector magnitude data revealed that some periods of non-movement were being classified as light intensity activity due to baseline error greater than the light activity cut-point. This influence of accelerometer error on activity classification led us to implement the accelerometer autocalibration procedure (see Implementation). In a subset of 479 device collections across 157 HANDDS-ONT participants (3–5 device collections per participant) on which autocalibration was performed, we found a mean (SD) accelerometer error of 29.0 (13.5) mg before calibration. For context, the light activity cut-points used for adults 60 years or older are 42.5 mg for the non-dominant arm and 62.5 mg for the dominant arm [47]. In this subset, 15.7% of device collections had error of at least 42.5 mg and 2.3% had error of at least 62.5 mg. Autocalibration reduced the mean (SD) error to 3.8 (1.3) mg.

#### Non-wear detection improvements

Early data review of potential non-wear periods revealed a characteristic change in near-body temperature when a device was removed and reattached, leading to development of our DETACH non-wear detection algorithm [38]. This novel algorithm considers standard deviation of acceleration, rate of temperature change, and absolute temperature over 5-min rolling windows to determine the start and end of non-wear periods. The values of these parameters were originally optimized for devices worn only at the wrist and further non-wear review revealed a disproportionate amount of non-wear misclassification for ankle- and chest-worn devices. As such, the same optimization procedure was performed to derive body location-specific parameters for devices worn at the chest and ankle and the option was added to specify custom parameters. Although our current non-wear methods are performing at, or better than, other published algorithms [38], differentiating non-wear from sleep or sedentary behaviour continues to pose challenges. For example, of the 81 HANDDS-ONT participants in whom non-wear was detected with body-location specific parameters, 34 (42%) required custom parameters to resolve instances of non-wear misclassification, usually overnight. Current development focuses on automatically generating personalized non-wear detection parameters based on the unique temperature and accelerometer data profile of each device worn by each participant. Meanwhile, ongoing expert data review remains focused on inspecting detected non-wear, sleep, and sedentary behaviour to ensure proper classification.

### Comparative analysis

It remains critically important to continue developing tools for wearables data analyses in health applications that output high-quality, fit-for-purpose data. Commercial and proprietary products are limited in their opportunity to support continued development and extended use for clinical purposes. To address this gap, a growing number of open-source analytics packages designed for use with a variety of wearable devices have emerged. Table 2 details open-source toolkits available to support analysis of wearables data. This list was compiled by conducting a scan of the literature as well as common data analytics repositories such as GitHub [52] and OWear [53]. Algorithms and toolkits were included if they met the following criteria: 1) they rely on access to raw sensor data, 2) they accommodate data collected for extended periods in daily life, outside of a lab or clinic, and 3) they generate outcomes that characterize daily health-related behaviors. Several open-source packages that provide support for signal processing of body worn sensors data were not included because they do not specifically characterize health-related behaviours during continuous wear. For example, BioPatRec [54] uses EMG for prosthetics control, Gait-tech [55] performs gait/motion analysis, OpenSignals [56] and Neurokit [57] provide both biosignal processing and visualization tools.

Most included packages focus on one specific domain (e.g., sleep or gait) or use data from a single node, most commonly the wrist, even when providing multi-domain outcomes (e.g., sleep, activity, mobility). This reliance on a single wrist node persists despite limitations of this location to assess whole body behaviours such as walking [51]. There is growing interest in and evidence supporting the feasibility and importance of multiple node locations [18, 21, 51], as used in Scikit Digital Health [70] and NiMBaLWear. An important additional advantage of NiMBaLWear is the embedded cross-device temporal synchronization, which provides the opportunity for comparing and fusing data from different nodes and modes. This capability enables upcoming releases of NiMBaLWear to include the option to fuse data from multiple modes (i.e., IMU and ECG). The benefit of an integrated, multi-domain platform is linked to the value of understanding the interplay between health-related behaviors such as the important relationships between sleep, physical activity, sedentary behavior, mood, social engagement, and symptoms of disease [24, 27, 71–75].

As highlighted in Table 2, most packages provide the option to import raw data from different devices or standard format data files (e.g., CSV or EDF), which affords access to common analytics regardless of which device(s) collected the data. This device independence facilitates access to raw sensor data that can be transparently transformed into measured outcomes of interest using open-source processing algorithms and pipelines. Further, a device-independent pipeline facilitates the multi-nodal, multi-modal approach required to derive multi-dimensional outcomes of interest, readily allows the inclusion of new and evolving hardware, and meets the imminent demand for increased scope and scale of wearables data analyses. Finally, in our experience, the flexibility of a device-independent pipeline has been paramount to meeting the challenges of developing and implementing remote clinical trials that must consider factors such as familiarity with a particular device, cost, concerns regarding validity of hardware and proprietary analytics [14], access to raw data, and device usability.

For the field of wearable technology to make strides in its application to healthcare, both transparency and validation of outcomes are vital. This includes the growing number of machine learning approaches that consolidate data from wearable devices to develop predictive models for healthcare decision-making. To this end, we recognize the importance of accessibility to both raw and tabular data for continued development of analytics, as well as full documentation for embedded analytics (e.g., error, run-time reporting). As noted in Table 2, most packages do provide the ability to visualize time series data and provide exported tabular data but the extent to which process outputs are documented varies. Further, NiMBaLWear is currently the only toolkit to produce outputs including standardized, accessible, and temporally synchronized raw data alongside data visualizations and a range of tabular data.

### Planned future developments and intended use

We have prioritized three areas for NiMBaLWear development to optimize uptake and utility. These priorities include: 1) improving wearable data quality before it is ingested into the pipeline, 2) leveraging sensor fusion techniques to add or improve domain- and disease-specific analytics, and 3) improving pipeline accessibility for those with limited programming expertise.

#### Data quality

Ongoing work aims to automatically set personalized non-wear detection parameters based on the unique temperature and accelerometer data profile of each device worn by each participant. The goal is to further reduce the frequency of misclassification of non-wear, sedentary, and sleep periods. We are also currently exploring the feasibility of synchronizing devices around naturally occurring behavior (e.g., walking) rather than relying on manual synchronization events during the collection period. While the current method is simple and robust, the proposed method will provide more frequent synchronization points while reducing participant burden.

#### Domain-Specific analytics

The addition of posture and ECG modules, as well as other expanded, sensor fusion-based analytics, are critical to maximizing the utility of wearables data for clinical application. Such advances will aid in resolving uncertainties in the data (e.g., sedentary activity classification from a wrist-worn IMU during walking) and improve evaluation of health-related behaviors. A beta version of the posture module, capable of classifying body segment orientation and full body posture, has been developed and is undergoing final stages of testing. While body segment orientation is derived from a single device, accurate full body posture classification requires fusion of accelerometer data from multiple synchronized devices worn on different nodes (e.g., chest and thigh). In addition to a posture module, future versions of NiMBaLWear will include a cardiac health module to derive meaningful outcomes from continuous ECG or heartbeat timeseries (e.g., via photoplethysmography (PPG)). While ECG (chest) and PPG (wrist) data are usually collected from a single device, fusion of these data with data from other modes and nodes will improve the quality of physiological and behavioural outputs related to circadian health, autonomic nervous system function, sleep detection, and activity intensity estimation. Sensor fusion-based analytics for gait and activity will also be made available in upcoming versions of NiMBaLWear. We have developed and are currently testing algorithms to detect gait from devices worn in various body locations. These algorithms will support gait detection from different sensor setups, improve detection and characterization of specific gait features, and help to refine cross-device synchronization.

#### Disease-Specific analytics

Given our interest in the use of wearables for clinical application, NiMBaLWear will continue expanding to include modules that serve specific purposes for our populations of interest. We are working to implement algorithms that target tremor, freezing of gait, upper limb impairment, and involuntary limb motion (e.g., periodic limb movement) experienced by some individuals with neurodegenerative disease. These modules will quantify symptom occurrence and severity in free-living and improve accuracy of gait, activity, and sleep detection from limb-worn IMUs for individuals with these symptoms. Additionally, in support of specific studies, we are beginning to develop and implement code that permits event marking (e.g., medication taking), as well as additional lifestyle-related modules targeting social engagement and GPS-based activity-space measures.

#### Pipeline and data accessibility

Our focus on accessibility of the pipeline and its outputs targets researcher and clinician end users who may not be skilled programmers and developers. This work includes efforts to optimize (and minimize) the sensors and data sets within our multi-sensor model. The need to determine the ideal parameters for a particular health application is important not only for reducing burden and maximizing feasibility, but also to inform industry partners as they continue to develop marketable solutions. Further, work is underway to develop a user-friendly interface for processing data through the pipeline to support those whose expertise is in the translation of such data (e.g., exercise prescription) and those who wish to explore potential applications of wearables outside their own areas of expertise. Lastly, within our group, advancing methods for information sharing is occurring alongside analytic and pipeline development to ensure successful integration of wearables-derived outcomes into workflows for self-management of health, clinical care, and shared clinical decision-making [49]. To this end, a reporting module has been developed to produce a pipeline output that transforms user-selected tabular data into a format that is easy for end users (i.e., clinicians, patients, care partners) to interpret. This module will be available within NiMBaLWear imminently, once flexibility to accommodate specific use cases has been integrated.

## Conclusion

With appropriate access to valid and reliable analytics, remote measurement using wearable sensors moves a critical step forward to becoming a viable and useful option for clinical decision-making. NiMBaLWear provides a flexible, open-source, wearable sensor analytic pipeline to transform raw multi-day, multi-sensor, free-living data into accurate and relevant outcomes across multiple health domains. Its iterative and incremental development using data from more than 250 participants ensures a robust and reliable product, and comparison of NiMBaLWear to other open-source packages demonstrates its unique capacity to derive, integrate, and share health-related behavioral outcomes from wearables data. Imminent improvements to the pipeline will further optimize wearable data quality, leverage sensor fusion techniques to refine and expand pipeline outputs and improve pipeline accessibility via a user-friendly graphical user interface. Conceptually, understanding an individual’s daily life behavior has the potential to transform clinical care and self-management of health. However, success depends on continued efforts within the field to establish relevant approaches and rigorous standards for the use of wearables-derived outcomes.

## Figures and Tables

**Table 1:** Domain-specific daily summary outcome measures

Domain	Outcome measure	Description
Device wear	Wear duration	Total duration device was worn while collecting data
	Non-wear duration	Total duration device was non worn while collecting data
Gait	Longest bout duration	Duration of the longest walking bout
	Longest bout steps	Number of steps in the longest walking bout
	Three-minute bouts	Number of walking bouts of three minutes or longer duration
	Total steps	Total number of steps
Activity	Sedentary duration	Total duration of sedentary (does not include sleep)
	Light activity duration	Total duration of light activity
	Moderate activity duration	Total duration of moderate activity
	Vigorous activity duration	Total duration of vigorous activity
Sleep^a^	Sleep window duration	Total duration of all SPTWs that contain detected sleep
	Sleep duration	Total duration of sleep detected
	Sleep-to-wake duration	Total duration from first sleep onset to last wake onset in all SPTWs that contain sleep
	Sleep efficiency	Decimal fraction of sleep duration relative to sleep window duration (Sleep duration / Sleep-to-wake duration)
	Wake duration after sleep onset	Duration of time spent awake between first sleep onset and last wake onset (Sleep-to-wake duration – Sleep duration)

a All sleep outcome measures are calculated and reported separately for the whole day (noon to noon) and overnight (10 pm to 8 am, by default)

**Table 2:** Comparison of open-source wearables analytics packages

Package Name (Code)	Domain(s)	Modes (Sensor Types)	Inputs (Specific Device/Data)	Nodes(Body Site)	Additional Notes
**Single domain - single device**					
SleepPy(Python) [58]	Sleep	Accel Temp Light	GENEActiv	Wrist	While the primary focus is sleep, does provide activity index Tables and charts for analysis and presentation of outputs and sleep measures for each day separately
GaitPy (Python) [59]	Gait	Accel	Formatted CSV	Lumbar	Uses single axis (vertical) from low back site and requires subject height Not currently maintained – Scikit Digital Health (listed below) includes a newer version of GaitPy
Verisense Toolbox Step Detection Algorithm (GGIR2.0) [60]	Gait	Accel	Same criteria as GGIR	Wrist	Add-on to GGIR for step detection Visualization of raw data Summary of step count data
GENEAclassify (R) [61]	Gait	Accel	GENEActiv	Wrist	Provides activity classification and intensity outcomes
Pampro (Python) [62]	Activity	Accel	Axivity GeneActiv Actigraph activPAL Formatted CSV	Wrist	Visualization of raw data Physical activity summary statistics Extract bouts for specific cut points
**Single domain – multiple devices**					
OpenSenseRT (Python and OpenSim) [63]	Limb motion	Gyro Accel	Raspberry Pi microcontroller Adafruit IMU	Many (up to 14)	Limb kinematics (flexion, extension, rotation, adduction) Visualizations and summary joint angles Requires hardware/component assembly
BDlab-OR: FoGdetection (Matlab) [10]	Gait	Gyro Accel	Formatted CSV	Lumbar Bilateral feet or shanks	Specific to detection of freezing of gait Import bouts of data containing gait activity Outputs number of freezing events
OpenIMU (Python) [64]	Activity	Accel GPS	Actigraph OpenlMU logger AppleWatch SensorLogger	Wrist	Visualization of time-series and summary activity metrics Export data as CSV or Matlab format
**Multiple domains – single device**					
GGIR (R) [65]	Sleep Activity Sedentary	Accel	GENEActiv Actigraph Axivity Movisens	Wrist	Visualization of data and tabular data across and within days Verisense (noted earlier) is a GGIR add-on for step detection
Biobank Accelerometer Analysis (Python) [66–69]	Sleep Activity Sedentary	Accel	GENEActiv Actigraph Formatted CSV	Wrist	Visualizations and data outputs within and across days Outputs time series of epoched data and bouts (CSV) Originally designed to use with UK Biobank data
**Multiple domains – multiple devices**					
Pfizer/Scikit-digital-health (Python) [70]	Sleep Gait Transition	Accel	GENEActiv Axivity APDM (Opal)	Lumbar Wrist	Specific wear locations (nodes) used for specific domains run separately (wrist for sleep and activity; low back for gait and transitions) Tables and charts for analysis and presentation (within and across days) Signal processing utilities
NiMBalWear (Python)	Sleep Gait Sedentary Activity	Accel Gyro Temp	Axivity GENEActiv Actigraph Bittium Formatted EDF	Wrist (uni- or bi-lateral Shank (uni- or bi-lateral), Trunk (sternum)	Temporal synchronization of all devices (based on standard synchronization protocol) allowing for time series comparison and fusion Modular design allows domains to be capture by different sensors (flexible sensor input set) Tables and charts for analysis and presentation within and across days Outputs resampled synchronized raw time series data (EDF)

Packages are categorized by number of health domain(s) addressed (one or multiple) and capacity for toolkit to process data from one (single node/mode) or more devices (multiple nodes/modes). (*Accel* accelerometer, *Gyro* gyroscope, *Temp* temperature, *GPS* global positioning system, *CSV* comma-separated values, *EDF* European data format)

## Data Availability

ReMiNDD and HANDDS-ONT data used for the development and evaluation of the NiMBalWear pipeline will be released through Brain-CODE (https://braincode.ca) by the OBI as part of the ONDRI IDP. Once released, data will be accessible upon reasonable request through a formal data access request. Please see the OBI website (https://braininstitute.ca) for information on timelines of release and the process for submitting a request. Inquiries regarding the subsets of data used to evaluate NiMBalWear performance can be directed to the corresponding author.

## Abbreviations

AVM: Average vector magnitude
CSV: Comma-separated values
DETACH: DEvice Temperature and Accelerometer CHange
ECG: Electrocardiography
EDF: European Data Format
GPS: Global Positioning System
HANDDS-ONT: Health in Aging, Neurodegenerative Diseases and Dementias in Ontario
IDP: Integrated Discovery Program
IMU: Inertial measurement unit
MVP: Minimum viable product
OBI: Ontario Brain Institute
ONDRI: Ontario Neurodegenerative Disease Research Initiative
ReMiNDD: Remote Monitoring in Neurodegenerative Disease
SPTW: Sleep period time window
SD: Standard deviation
